# Glucose and Fructose Transport Across the Epitheliochorial Placenta: SLC2A and the Uterine-Placental Interface in Pigs

**DOI:** 10.1210/endocr/bqaa138

**Published:** 2020-11-04

**Authors:** Daniel J Mathew

**Affiliations:** Department of Animal Science, University of Tennessee, Knoxville, TN

For pigs and other mammals, the first month of gestation is the most critical for survival of potential offspring. During the first 2 weeks of development, early pig conceptuses (embryo and extraembryonic tissues) remain unattached to the uterine luminal epithelium (LE) and are bathed in a milieu of uterine secretions collectively referred to as uterine histotroph (1). Largely produced by the uterine glandular epithelium in response to progesterone (P4), histotroph is composed of proteins, steroids, prostaglandins, ions, and hexose sugars, glucose and fructose, that support growth and development of the conceptus (1).

On approximately day 12 of development, pig conceptuses rapidly elongate in structure due to reorganization of the trophectoderm and subsequently expand within the uterine lumen (2). Elongation increases surface area of the conceptus to release important signaling molecules, such as estrogens, onto the uterine surface and increases the size of the future placenta (1). Conceptus estrogens have essential functions during pregnancy in pigs including maintenance of ovarian P4 secretion from corpora lutea (1).

Pig conceptuses attach to the uterine LE between days 13 and 18 of development (3). Although the trophectoderm send long cytoplasmic projections between LE cells, they do not invade beyond LE tight junctions, resulting in formation of an epitheliochorial placenta with as many as 6 cell layers between uterine and placental blood (3). By day 40 of gestation, important structural modifications occur along the uterine-placental interface that aid in absorption of maternal nutrients. These include chorionic villi and areolae; the latter are large dome-like structures that form over uterine glands and absorb histotroph (1).

In animals with an epitheliochorial placenta, maternal blood glucose is transported across multiple cell layers to reach the developing conceptus or placental capillaries. Facilitated diffusion of glucose and fructose across the cell plasma membrane involves solute carrier (SLC) transporters of the solute carrier 2A (SLC2A; GLUT) and 5A (SLC5A; SGLT) family. Although characterized in animals with hemochorial placentae, such as humans and rodents, uterine and placental expression of glucose transporters SLC2A1-4 have not been characterized in pigs, a species with a true epitheliochorial placenta (4, 5). Studies in sheep suggest that expression of uterine SLC2A transporters are influenced by ovarian P4 during the estrous cycle and early pregnancy (6). Further, the discovery that the pig endometrium and placenta convert glucose to fructose, which is then sequestered within fetal tissues at high concentrations, has raised questions regarding the function of this hexose sugar during pregnancy in pigs (7).

A recent study published in *Endocrinology* by Kramer et al (2020) teases apart the complex biology surrounding SLC2A1-4 transporter expression along the pig uterine-placental landscape and hexose sugar (glucose and fructose) metabolism in early pig conceptuses. Pig intrauterine glucose concentrations are first measured between days 9 and 15 of the estrous cycle and early pregnancy. Intrauterine glucose concentrations increased with the day but were not affected by pregnancy. Pig conceptus tissues were then incubated with combinations of isotope- and non-isotope-labeled glucose and fructose, quantifying conceptus hexose metabolism by measuring liberated carbon dioxide. Here it was noted that the conceptus tissues metabolized both glucose and fructose, yet preferentially oxidized glucose. Fructose metabolism increased when glucose was omitted.

In an effort to characterize uterine and placental cell-specific expression of glucose transporters, Kramer et al assayed cyclic and pregnant pig uterine tissues for SLC2A1-4 using a combination of quantitative polymerase chain reaction, in situ hybridization (ISH), and immunofluorescent (IF) techniques. Patterns in expression were compared with patterns in endometrium collected from ovariectomized pigs treated with P4 and intact pigs treated with estradiol (E2) to elucidate the effects of ovarian P4 and conceptus estrogens, respectively, on endometrial SLC2A.

Results from these experiments exposed the complex spatiotemporal pattern of SLC2A1-4 transporters along the uterine-placental interface in pigs and their regulation by steroid hormones P4 and E2. The spatial pattern was clearly evident in ISH and IF images of glucose transporters SLC2A3 and SLC2A1 in chorionic villi and areolae epithelium (Figures 3, 5, and 8). Modes of maternal glucose transport between hemochorial and epitheliochorial placentae are compared, highlighting the role of glucose and fructose transporters, SLC2A1-4 and SLC2A8, respectively, in hexose sugar diffusion between the maternal and fetal vasculatures. In Figure 9, Kramer et al break down the pig uterine-placental interface and depict the hypothesized diffusion of glucose and fructose through each cell type and specific SLC2A transporter between maternal and fetal vasculatures. Figure 10 depicts the spatiotemporal pattern of SLC2A transporter expression along the pig uterine-placental interface between days 15 and 60 of gestation.

In conclusion, Kramer et al show that transport of glucose between maternal and fetal vasculatures in the pig involves spaciotemporal patterns in expression of SLC2A1-4 transporters along the uterine-placental interface. Ovariectomy and pseudopregnancy experiments suggest these patterns are partially controlled by ovarian P4 and conceptus estrogens, coordinating efficient glucose transport from mother to placenta. Additional research is needed to elucidate the mechanism by which specific regions of the endometrial or chorionic epithelia have different SLC2A expression profiles. Importantly, glucose is actively converted to fructose within the pig placenta. Previous studies suggest fructose can enter the hexosamine biosynthesis pathway and stimulate trophectoderm proliferation via activation of the mechanistic target of rapamycin (8). Recently, it was discovered that pig conceptuses express enzymes that would allow fructose to enter the glycolytic pathway, suggesting its utilization by the conceptus as an alternative energy source (7). The observation made by Kramer et al that pig conceptus tissues oxidize fructose further supports this notion and expands our understanding of conceptus and fetal metabolism in pigs, a species critical for the production of food and disease research.

## Data Availability

***Data Availability:*** Data sharing is not applicable to this article as no datasets were generated or analyzed during the current study.

## Abbreviations

E2: estradiol
IF: immunofluorescent
ISH: in situ hybridization
LE: luminal epithelium
P4: progesterone
SLC: solute carrier

## Additional Information

***Disclosure Summary:*** The author has nothing to disclose.
